# Changes in the MALT1-A20-NF-κB expression pattern may be related to T cell dysfunction in AML

**DOI:** 10.1186/1475-2867-13-37

**Published:** 2013-04-30

**Authors:** Li Shi, Shaohua Chen, Yuhong Lu, Xu Wang, Ling Xu, Fan Zhang, Lijian Yang, Xiuli Wu, Bo Li, Yangqiu Li

**Affiliations:** 1 Institute of Hematology, Jinan University, Guangzhou, 510632, China; 2 Key Laboratory for Regenerative Medicine of Ministry of Education, Jinan University, Guangzhou, 510632, China; 3 Guangdong Province Key Laboratory of Molecular Immunology and Antibody Engineering, Jinan University, Guangzhou, 510632, China

**Keywords:** MALT1, A20, NF-κB, T cells, Acute myeloid leukemia

## Abstract

To elucidate the characteristics of T-cell receptor (TCR) signal transduction in T-cells from acute myeloid leukemia (AML), the mucosa-associated-lymphoid-tissue lymphoma-translocation gene 1 (MALT1), A20, NF-κB and MALT1-V1 gene expression levels in CD3^+^ T cells sorted from the peripheral blood of patients with AML were analyzed by real-time PCR. A significantly lower MALT1 and A20 expression level was found in T cells from patients with AML compared with healthy controls (*p* = 0.045, *p* < 0.0001); however, the expression level of MALT1-V1 (variant 1) was significantly higher in the AML group than in the healthy control group (*p* = 0.006), and the expression level of NF-κB was increased in the AML group. In conclusion, the characteristics of the expression pattern of MALT1-A20-NF-κB and the distribution of MALT1 variants in T cells from AML were first characterized. Overall, low TCR-CD3 signaling is related to low MALT1 expression, which may related to T cell immunodeficiency, while the up-regulation of MALT1-V1 may play a role in overcoming the T cell activity by downregulating A20 in patients with AML, which may be related to a specific response to AML-associated antigens.

## Background

Acute myeloid leukemia (AML) is the most common acute leukemia affecting adults, and its incidence is expected to further increase as the population ages. An important approach for prolonging remission duration and eradicating minimal residual disease in leukemia is immunotherapy. The therapeutic value of donor lymphocyte infusions (DLI) in patients who relapse with AML is limited by a low efficacy and a high risk of GVHD. All of these attribute to the T cell mmunodeficiency. The development of AML is a multistep process that requires at least two genetic abnormalities for the development of the disease [1-3]. Moreover, cell-mediated immunity is often suppressed in patients with hematological malignancies with T cell dysfunction being the most profound for those with advanced disease [4-8]. And T cell immunodeficiency in patients with AML is more obvious. T cell immunodeficiency in AML is characterized by a low recent thymic output function [9,10], abnormal T cell receptor (TCR) repertoire distribution [11], and a lower ability to form effective immunologic synapses with autologous AML blasts [4]. Recently, we found an abnormal expression profile of genes related to TCR signaling e.g., CD3ζ, in T cells from AML [4]. In addition, it was found that low CD3ζ expression is significantly involved in deficiencies in T cell activation, which could be rescued after enhancing CD3ζ expression by gene modification [12]. It is interesting to further investigate the molecular characteristics of T cell dysfunction in AML.

A20 (also known as TNFAIP3) was first discovered in 1990 as a cytokine-induced gene in human umbilical vein endothelial cells [13]. This protein is a dual ubiquitin-editing enzyme involved in the termination of nuclear factor-κB (NF-κB) signaling [14-17]. A20 regulates innate and adaptive immunity, and T cells express high basal levels of A20, which decrease upon T-cell activation [18].

Recent data have demonstrated that A20 plays a crucial role in B and T cell signaling activation. A20 is commonly deleted in several subtypes of B-cell lymphomas (approximately 40%) including marginal zone, diffuse large B-cell, follicular, MALT and Hodgkin lymphomas. The loss of A20 has also been found in Sezary syndrome and cutaneous T-cell lymphoma [14,15,19]. A20-silenced DCs demonstrated spontaneous and enhanced expression of costimulatory molecules and proinflammatory cytokines and had different effects on T cell subsets i.e., they inhibited Treg cells, hyperactivated tumor-infiltrating cytotoxic T lymphocytes and T helper cells that produced interleukin-6 and tumor necrosis factor-α and were refractory to Treg cell–mediated suppression [20,21].

The NF-κB transcription factor is a chief regulator of lymphocyte activation, survival and proliferation. The signal transduction pathways initiated by the TCR and B cell antigen receptor (BCR) lead to NF-κB activation. TCR and BCR triggering leads to the activation of the serine-threonine kinases PKC-θ and PKC-β, respectively, which subsequently phosphorylate the caspase-recruitment domain (CARD)–containing membrane-associated guanylate kinase protein 1 (CARMA1; also called CARD11) [22]. This phosphorylation event facilitates the recruitment of the CARD containing adaptor protein B-cell lymphoma 10 (Bcl-10) and the paracaspase mucosa-associated-lymphoid-tissue lymphoma-translocation gene 1 (MALT1), resulting in assembly of the CARMA1–Bcl-10–MALT1 (CBM) complex. Studies have demonstrated the essential function of MALT1 in TCR signaling, and upon TCR engagement, human A20 is cleaved by MALT1 after arginine 439, yielding N-terminal (hA20p50) and C-terminal (hA20p37) fragments. These observations emphasize the importance for understanding MALT1 mediated rapid proteolytic cleavage and inactivation of the NF-κB inhibitor A20 after TCR stimulation. Moreover, MALT1 is thought to be a possible target for the development of immunomodulatory or anticancer drugs [22,23].

A20 mutations and polymorphisms resulting in decreased A20 expression were found to be associated with autoimmune diseases such as SLE and RA [24,25]. Little is known about the role of A20 and its related genes MALT1 and NF-κB in T cells in patients with AML. Based on our previous finding of low CD3 gene expression in T cells from patients with AML, in this study, we analyzed the expression level of the MALT1, A20 and NF-κB genes and found that an alternative expression pattern for these genes may be related to T cell dysfunction in AML.

## Results and discussions

Recently, it has become evident that triggering the TCR controls T-cell proliferation through proteases such as MALT1. These proteases, which are relevant to the control of the T-cell response, represent interesting targets for therapeutic immunomodulation [26] and may enhance immunity in patients with leukemia. Our previous findings demonstrated low expression of the CD3γ, δ, ϵ and ζ chain genes in T cells sorted from peripheral blood mononuclear cells from patients with AML [4]. However, clonally expanded T cells were found in patients with AML, which may indicate T cell immune activation and a response to AML-associated antigens [9]. To investigate the downstream gene expression characteristics of the TCR-CD3 pathway, the expression level of the MALT1, A20 and NF-κB genes was determined in the same CD3^+^ T cell samples by real-time PCR.

**Figure 1:**
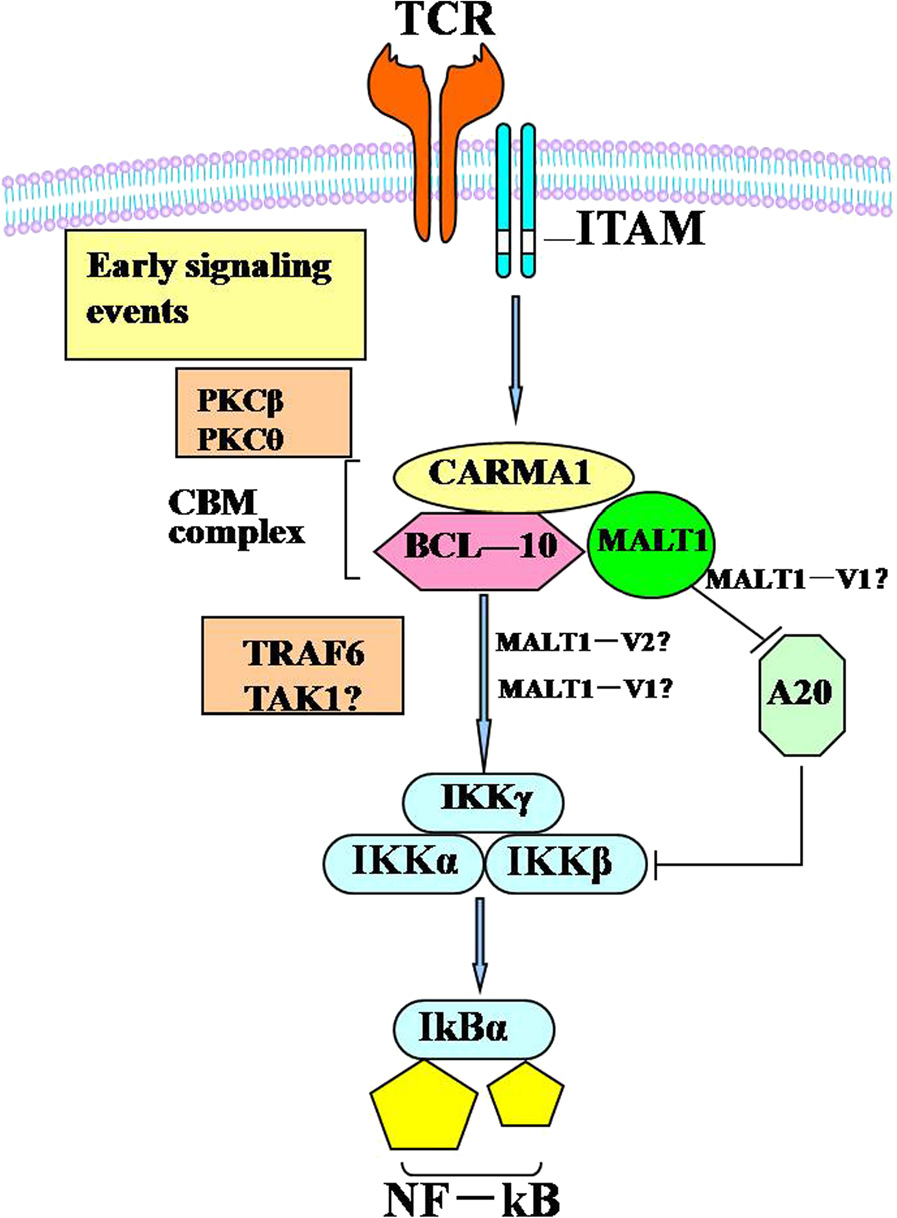
**Schematic diagram of signal transduction of MALT1-A20-NF-**κ**B in T cells from patients with AML.**

T cell activation was triggered by the T-cell receptor and CD3 complex. Recent findings define MALT1 as a protein with proteolytic activity that controls T-cell activation by regulating key molecules in TCR-induced signaling pathways. CARMA1, BCL&nonBR;10 and MALT1 act together in the activation of NF-κB downstream of CD3 chains containing immunoreceptor tyrosine-based activation motifs (ITAMs). Activation of the proteolytic activity of MALT1 is thought to contribute to NF&nonBR;κB activation through the proteolytic degradation of the NF&nonBR;κB inhibitor A20 (Figure 1) [27].

**Figure 2:**
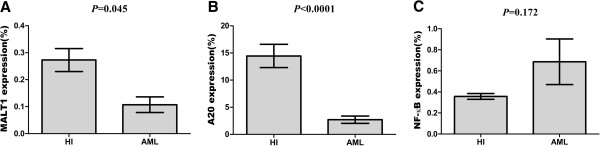
**The relative expression level of the MALT1 (A), A20 (B), and NF-**κ**B (C) genes in the healthy control and AML groups.**

Based on the regulatory role of MALT1 and A20 in T cell signaling activation, we first analyzed the expression level of MALT1 and A20 in T cells from patients with AML. Significantly lower expression levels of MALT1 in T cells was found in the AML (0.11 ± 0.09) compared with the healthy group (0.27 ± 0.13) (*p* = 0.045) (Figure 2A), which may be associated with a lower activation of T cells in AML; however, interestingly, the expression of A20 was lower in the AML group (2.71 ± 2.14) than that in the healthy controls (14.45 ± 6.42) (*p* < 0.0001) (Figure 2B). Because A20 acts as a negative regulatory factor in the activation of T cells, both results were not in accordance. Therefore, we further analyzed the expression of NF-κB in T cells, and the results demonstrated that its expression level was high in the AML group (0.69 ± 0.22) in comparison with the healthy group (0.36 ± 0.03); however, the difference was not statistically significant (*p* = 0.172) (Figure 2C). Based on the findings of NF-κB in T cells from AML, it is thought that activation occurred in at least a portion of the T cells from AML, and this activation may be directly regulated by A20, while low MALT1 could directly down-regulate the expression of NF-κB (Figure 1) [27], which may explain the phenomenon of low MALT1 and A20 levels with moderate increasing NF-κB. However, this explanation may be limited due to the relatively small sample size, further investigation is needed. Lower A20 expression levels have been found in most autoimmune diseases, such as SLE and RA [24,25], and are associated with enhanced expression of costimulatory molecules and proinflammatory cytokines [20,21]. In contrast, the decreased A20 in T cells from patients with AML may be due to the activation of a subset of T cells, which is thought to be a specific response to AML cells, and the finding of clonally expanded T cells from AML and other leukemias may support this hypothesis [9,28-30].

**Figure 3:**
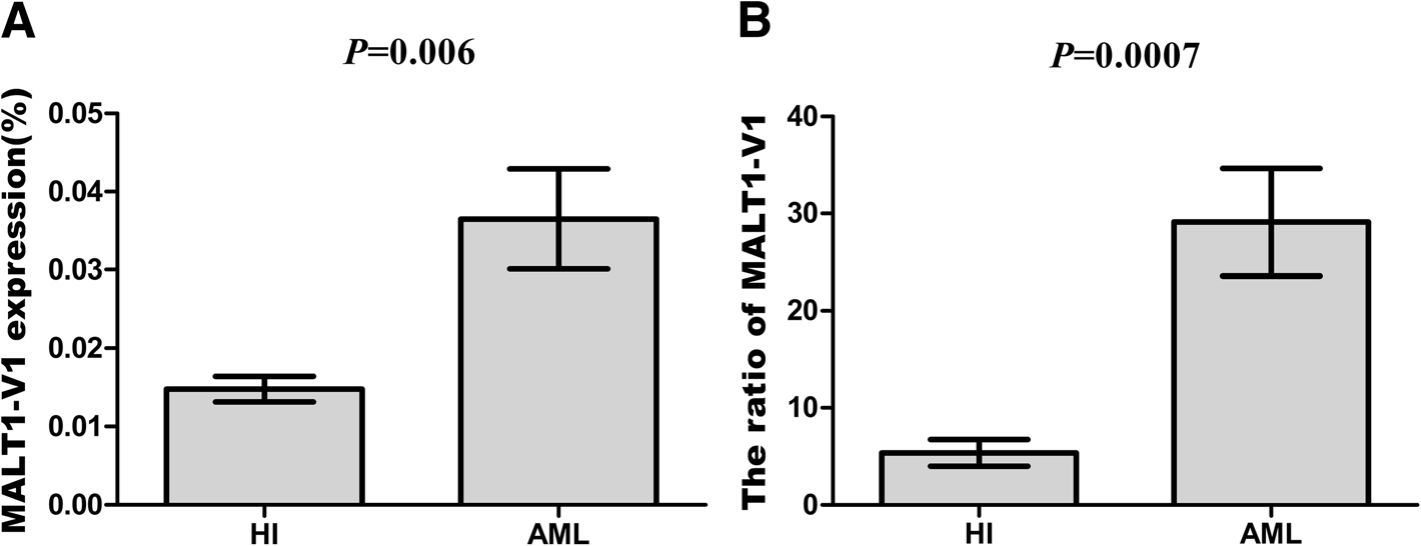
The relative expression level (A) and ratio (B) of the MALT1-V1 gene in the healthy control and AML groups.

To gain more insight into the characterization of MALT1 in the regulation of T cell activation, we further analyzed the distribution of MALT1 isoforms. Our previous study found that MALT1 variants (MALT1-V1 and MALT1-V2) could be identified by RT-PCR and sequencing (data not shown). In this study, we found that the MALT1-V1 expression level was significantly higher in the AML (0.04 ± 0.02) compared with the healthy control group (0.01 ± 0.005) (*p* = 0.006) (Figure 3A). Because we could not directly amplify MALT1-V2, which contains a 33 bp deletion, the expression level of MALT1-V2 could only be indirectly calculated as the relative expression of MALT1-V1/total MALT1, it is implicit that the MALT1-V2 expression level was downregulated in T cells from patients with AML.

There are no previous reports describing the expression pattern, distribution, or different biological functions of the MALT1 variants in the literature, and here we report for the first time the change in the expression pattern of MALT1 variants in T cells from patients with AML, including not only the expression level of MALT1-V1 but also the ratio of MALT1-V1 to the total amount of MALT1 (Figure 3B), which was significantly upregulated, suggesting that the MALT1 variants may have different biological functions or different targets. The combination of the A20 and NF-κB expression pattern results suggests that MALT1-V1 may target A20 (Figure 1); therefore, higher MALT1-V1 expression results in decreased A20 levels in T cells from patients with AML. However, a significant negative correlation could not be found between the expression levels of MALT1-V1 and A20, which may be due to the limited number of samples, and further study analyzing more samples is needed. Moreover, no information exists regarding NF-κB regulation by different MALT1 variants, and whether MALT1-V1, MALT1-V2 or both regulate the NF-κB-regulating IKK complex (Figure 1) [27], remains an open question.

In conclusion, in this study, we described for the first time the characteristics of the MALT1-A20- NF-κB expression pattern and the distribution of MALT1 variants in T cells from patients with AML. Overall, low TCR-CD3 signaling was related to low MALT1 expression, which may be related to T cell immunodeficiency, while the upregulation of MALT1-V1 may play a role in overcoming the activity of T cells in AML by downregulating A20, which may be related to a specific response to AML-associated antigens. Further investigation is required to analyze the detailed functions of MALT1-V1 and MALT1-V2 in T cells, which is important for the characterization of the molecular mechanisms of T cell activation and dysfunction in AML.

## Materials and methods

### Samples

The samples used in this study were derived from ten newly diagnosed, untreated patients with de novo AML including seven males and three females (13–65 years old; median age: 36 years) [4]. Peripheral blood mononuclear cells (PBMCs) were isolated from heparinized venous blood by Ficoll-Paque gradient centrifugation. CD3^+^ T cells were sorted from the ten patient samples and nine healthy individuals using anti-CD3 monoclonal antibodies and the MACS magnetic cell sorting technique (Milten Yi Biotec, Germany). Ten healthy individuals including five males and four females (24–54 years old; median age: 38.5 years) served as controls. All procedures were conducted according to the guidelines of the Medical Ethics Committee of the Health Bureau of the Guangdong Province in China.

### Primer design and real-time quantitative reverse transcription-polymerase chain reaction (qRT-PCR)

The sequences of the primers for MALT1, A20 and NF-κB gene amplification are listed in Table 1. According to the structure of the MALT1 gene, there are two variants, named MALT1-V1 and MALT1-V2, and the latter contains a 33 bp deletion located between exons 6 and 8. To amplify the two MALT1 transcript variants, the primer pair MALT-V1-F and MALT-V1-R was designed for MALT1-V1 amplification to cover the region that MALT1-V2 is missing, and the primer pair MALT1-F and MALT1-R was designed to amplify the conserved region, which is contained by both variants. The expression level of the A20, MALT1, MALT1-V1, NF-κB and β2-microglobulin (β2M) genes was determined by SYBR Green I real-time PCR. Briefly, PCR was performed in a 20 μL total volume that contained 1 μL of cDNA, 9 μL of 2.5× SYBR Green I mix (Tiangen, Beijing, China), and 10 μmol/L primer pairs. After an initial denaturation at 95°C for 15 min, 45 cycles consisting of the following procedure was performed using an MJ Research DNA Engine Opticon 2 PCR cycler (BIO-RAD, USA):30 s at 95°C, 40 s at 60°C for β2M and A20, MALT1, MALT1-V1, NF-κB [6]. The relative amount of the genes of interest and the β2M reference gene was measured in two independent assays. Specific amplification of the PCR products was analyzed by melting curve analysis. The data are presented as the relative expression of the genes of interest compared to the internal control gene as determined by the 2(^-△CT^) method [6]. In addition, to analyze the MALT1-V1 expression features, we calculated the MALT1-V1 expression ratio as MALT1-V1/MALT1 × 100%.

**Table 1:** Primer sequences used for real-time RT-PCR

**Primers**	**Sense and Antisense**	**Accession no.**	**Products**
A20F	5′-CTGGGACCATGGCACAACTC-3′	NM006290	182 bp
A20R	5′- CGGAAGGTTCCATGGGATTC -3′		
MALT1F	5′-TCTTGGCTGGACAGTTTGTGA-3′	NM_006785.2	230 bp
MALT1R	5′-GCTCTCTGGGATGTCGCAA-3′		
NF-κBF	5′-CCACAAGACAGAAGCTGAAG-3′	NM003998	149 bp
NF-κBR	5′-AGATACTATCTGTAAGTGAACC-3′		
MALT1-V1F	5′-AAGCCCTATTCCTCACTACCAG-3′	NM_006785.2	195 bp
MALT1-V1R	5′ -CACTCCACTGCCTCATCTGTTC-3′		
*β*_2m_F	5′- TACACTGAATTCACCCCCAC -3′	J00105	145 bp
*β*_2m_R	5′- GCGGCATCTTCAAACCTC -3′		

### Statistical analysis

Differences in the mRNA expression between two groups were analyzed using the unpaired Student’s *t*-test. The data are presented as the mean ± SD. Differences with a *P* < 0.05 were considered statistically significant.

## Competing interests

The authors have no potential conflicts of interest.

## Authors’ contributions

YQL contributed to the concept development and study design. LS, SHC, XW, LX and FZ performed the real-time PCR and LJY and XLW performed the CD3 + T cell sorting. YHL and BL were responsible for the collection of clinical data. YQL and SL coordinated the study and helped draft the manuscript. All authors read and approved the final manuscript.
